# Modulation effect of acupuncture treatment on chronic neck and shoulder pain in female patients: Evidence from periaqueductal gray‐based functional connectivity

**DOI:** 10.1111/cns.13803

**Published:** 2022-01-19

**Authors:** Hui Xu, Yilin Chen, Yin Tao, Yiwen Zhang, Teng Zhao, Mi Wang, Lihua Fan, Yunsong Zheng, Chenguang Guo

**Affiliations:** ^1^ Department of Medical Imaging The First Affiliated Hospital of Xi’an Jiaotong University Xi’an China; ^2^ Department of Psychiatry and Behavioural Neurosciences McMaster University Hamilton Canada; ^3^ Department of Acupuncture Affiliated Hospital of Shaanxi University of Chinese Medicine Xianyang China; ^4^ Department of Radiology Affiliated Hospital of Shaanxi University of Chinese Medicine Xianyang China; ^5^ Department of Ultrasound Xi’an Gaoxin Hospital Xi’an China

**Keywords:** acupuncture treatment, chronic neck and shoulder pain, functional connectivity, periaqueductal gray, posterior insula

## Abstract

**Aims:**

Chronic neck and shoulder pain (CNSP) is a common neurological disorder, which females are more likely to suffer from. The periaqueductal gray (PAG) plays a key role in the descending modulation of pain. This study aimed to investigate altered PAG‐based functional connectivity (FC) in female patients with CNSP related to healthy controls (HCs) and the effect of acupuncture for female patients with CNSP using PAG‐based FC biomarkers.

**Methods:**

PAG‐based FC value was calculated based on resting‐state functional images and then compared between patients with CNSP at pre‐acupuncture, post‐acupuncture, and HCs. Then, correlational analyses were performed to examine the relationships between increased PAG‐based FC strength and improved clinical parameters in patients after acupuncture treatment.

**Results:**

Before acupuncture treatment, compared to HCs, patients with CSNP showed altered PAG‐based FC with widely distributed brain regions, including the left medial superior frontal gyrus, bilateral posterior insula (pIns), and cingulate gyrus. After treatment, patients with CNSP exhibited specially improved PAG‐pIns FC compared to that before treatment, and no significant difference was observed in the increased PAG‐pIns FC strength between HCs and patients with CNSP after treatment. Furthermore, pain catastrophizing reduction was significantly correlated with the increased PAG‐pIns FC strength in patients after treatment.

**Conclusion:**

The effect of acupuncture treatment may relate to the increased PAG‐pIns FC, which significantly correlated with pain catastrophizing reduction after treatment. These findings shed important mechanistic information on the role of therapeutic approaches in treating chronic neck and shoulder pain.

## Graphical abstract

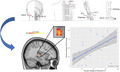

## 1 INTRODUCTION

Globally, chronic pain has a prevalence of 12%–30%^1^, ^2^, ^3^ and is related to the limitation of movement and daily activities,^4^ which seriously affects mental health^5^ and causes a lower quality of life.^4^ Clinical evidence showed that there were sex differences in the likelihood of developing pain‐related disorders,^6^ especially the incidence of fibromyalgia of chronic shoulder and neck pain (CNSP) in females, which was significantly higher than that in males.^7^ Females were found more sensitive to pain and exhibited a lower pain threshold and toleration than males.^8^ Therefore, females were more likely to be troubled CNSP, from which the adverse effect lasted longer.^8^

An increasing body of magnetic resonance imaging (MRI) literatures showed that individuals with chronic pain exhibited brain structural and functional alterations, which were related to pain.^9^, ^10^, ^11^ Additionally, previous clinical studies have found that individuals with chronic pain exhibited obvious clinical symptoms, such as depressive symptomatology, unsensitive sensory, and impaired information processing.^12^, ^13^, ^14^, ^15^ Similarly, several studies have found individuals with CNSP had abnormal neuronal activity in widely distributed brain regions, which involved in the integration and processing of pain signals, including the bilateral middle frontal gyrus, left insula, superior frontal gyrus, middle cingulate gyrus, and superior parietal lobule.^16^, ^17^, ^18^

Acupuncture is currently one of the main methods for the treatment of CNSP.^18^, ^19^, ^20^ There is increasing evidence, suggesting that its curative effect is considerable, and the therapeutic effect can last for at least 1 year.^20^, ^21^, ^22^ One study has aimed to investigate whether the acupuncture treatment can modulate brain abnormalities in CSNP using regional homogeneity (ReHo), and found that the alleviation of symptoms after treatment can normalize the reduced ReHo, which was significantly associated with a reduction in neck pain intensity.^23^ Additionally, another clinical study found that acupuncture treatment can reduce CNSP and related headache in female workers, and this long‐term effect may last for at least 3 years.^18^ Despite this, a few studies have examined whether acupuncture may be effective for female patients with CNSP using neuroimaging biomarkers.

Previous studies have found that functional connectivity between the pain‐related brain regions and periaqueductal gray (PAG) was modulated during acupuncture.^24^, ^25^ A large body of research suggested that the PAG plays a vital role in both the classic spinothalamic pain pathway and the emotional parabrachial pain pathway,^26^ which is crucial role for pain behavior forming.^25^, ^27^ If acupuncture treatment can recover the abnormal PAG functional connectivity patterns in CNSP, the pain behavior would be unlearned; finally, CNSP could be reversed.^27^ This led us to investigate whether acupuncture treatment could be involved in normalizing PAG functional circuitry in female patients with CNSP. Furthermore, previous research suggests that posterior insula (pIns) is both anatomically and functionally well suited to serve a primary and fundamental role in pain processing,^28^, ^29^ and individuals undergoing painful experiences were found to consistently show activation of pIns, which was with high correlation with pain intensity.^9^, ^30^, ^31^, ^32^ Another study has found that the functional connectivity between PAG and insula could predict perceived painfulness.^33^ This may led us to examine the specific PAG‐pIns functional connectivity would show significant changes in patients after acupuncture treatment.

Thus, the main purpose of this study was to examine whether acupuncture treatment can reduce neck pain and improve neck function in female patients with CNSP. A further aim was to see whether acupuncture treatment can modulate abnormal PAG functional connectivity patterns in CNSP. Therefore, the present study combined resting‐state functional MRI and acupuncture treatment to explore modulation effect of acupuncture treatment on CNSP. We hypothesized that (1) patients with CSNP showed altered PAG‐based functional connectivity with widely distributed brain regions compared with healthy controls (HCs) before acupuncture treatment; (2) after treatment, patients with CNSP exhibited improved PAG‐pIns functional connectivity compared to that before treatment, and there was no significant PAG‐pIns functional connectivity level difference between HCs and patients with CNSP after treatment; (3) after treatment, pain catastrophizing reduction was significantly correlated with the increased PAG‐pIns functional connectivity strength in patients with CNSP.

## 2 MATERIALS AND METHODS

### 2.1 Participants

Thirty right‐handed patients with CNSP (all females, mean age 42.33 ± 13.16 years old) were recruited from Department of Acupuncture at Affiliated Hospital of Shaanxi University of Chinese Medicine. The diagnosis of CNSP was assessed by two experienced clinical pain experts (who were blinded to this study) according to the classification of chronic pain for International Classification of Diseases 11th Revision (ICD‐11).^34^ The inclusion criteria were listed below: (1) complaints of neck and shoulder pain (with or without radiating arm pain) for at least 6 months with cervical degeneration in X‐ray and MRI; (2) no pain in other parts of body; and (3) not receiving any treatment within the last one month before this study. The exclusion criteria were (1) history of neck trauma; (2) vertebral body or spinal canal cancer, tuberculosis, or severe osteoporosis; (3) history of neck surgery or presence of congenital malformation of the cervical vertebrae; (4) pregnant or breastfeeding; and (5) presence of a severe systemic disease such as tumors, or digestive system disease; and (6) MRI contraindications. Additionally, 30 pain‐free age‐ and sex‐matched HCs were recruited from the same geographic area by public advertisement. All HCs also met the above exclusion criteria.

This study has been approved by the local ethics review board. All participants provided written informed consent according to the Declaration of Helsinki after study procedures were explained to them thoroughly. This study was registered at https://www.chictr.org.cn/index.aspx (ChiCTR2000030383).

### 2.2 Neuropsychological assessment

A series of neuropsychological measures were conducted to assess pain intensity, neck functionality, and clinical symptoms in all patients with CNSP at pre‐treatment (within one day before the first treatment) and post‐treatment (within one day after the final treatment). Numerical Rating Scale (NRS) was performed to rate the extent of pain within recent one week. Pain Catastrophizing Scale (PCS) was used to measure trait pain catastrophizing. The Northwick Park Neck Pain Questionnaire (NPQ) was administered to assess neck pain and disability.^19^ The Neck Disability Index (NDI) was also conducted to identify self‐reported neck function.^15^ Additionally, Montreal Cognitive Assessment (MoCA) and Mini‐Mental State Examination (MMSE) were performed to evaluate memory, attention, and executive function. The level of anxiety and depression of all participant was examined by Hamilton Anxiety Rating Scale (HAM‐A) and Hamilton Depression Rating Scale (HAM‐D).^35^

In addition, only MoCA, MMSE, HAM‐A, and HAM‐D assessments were performed once in all pain‐free HCs. All neuropsychological assessments were conducted and reviewed by an experienced clinical psychologist, who was blinded to the experimental procedures.

### 2.3 Acupuncture treatment

**FIGURE 1:**
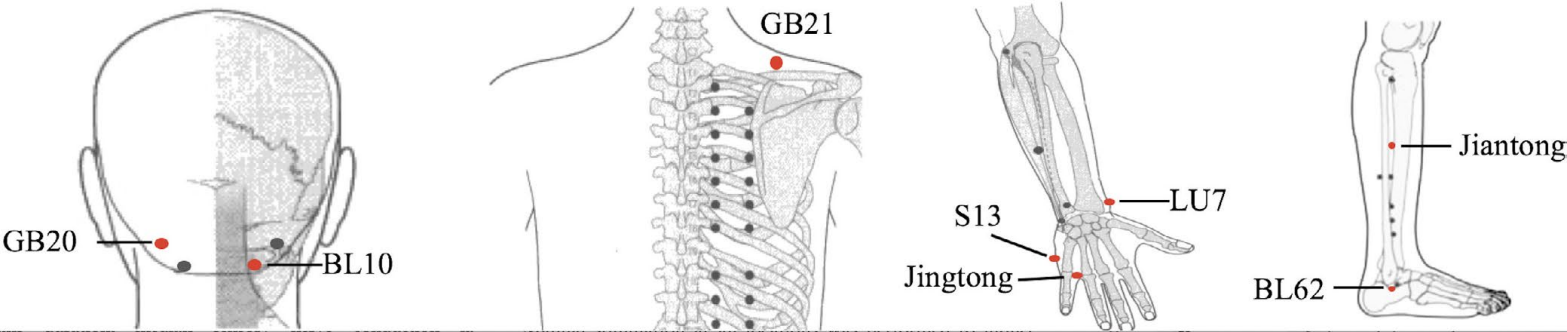
Location of acupoints for acupuncture treatment: GB20, GB21, S13, LU7, Jingtong, Jiantong, and BL62. Acupoints where stimulation was provided via needles were marked as red points

All patients with CSNP received 20 acupuncture treatments for four weeks at Department of Acupuncture at Affiliated Hospital of Shaanxi University of Chinese Medicine. Acupuncture was delivered to patients in supine position by a trained, licensed acupuncturist with at least 5 years of clinical experience, who was blinded to this study. During acupuncture treatment, acupuncture needles were placed at acupoints GB20, GB21, S13, LU7, Jingtong, Jiantong, and BL62 (Figure 1 for all acupoint locations).

Each patient with CNSP received about 30 mins of acupuncture treatment approximately once every day for a total of 20 sessions over a four‐week period. The needles were inserted to a depth of 20–30 mm, perpendicular to the surface of the skin. Acupuncture was performed to stimulate the relevant acupoints for 30 min using a comfortable strength until deqi sensation (soreness, numbness, distention, and heaviness) was obtained.^23^

### 2.4 MRI data acquisition

Neuroimaging data from patients with CNSP were acquired twice at pre‐treatment (within one day before the first treatment) and post‐treatment (within one day after the final treatment) on a 3T whole‐body MR system (Magnetom Skyra, Siemens, Germany) with a circularly polarized 32‐channel matrix head coil. The head of each participant was snuggly fixed with foam pads to reduce head movements and scanner noise. For each participant, a T1‐weighted high‐resolution structural image was acquired using magnetization prepared rapid acquisition gradient echo sequence with the following parameters: field of view (FOV) =256 × 256 mm^2^, matrix size = 256 × 256, time of repetition (TR) = 2300 ms, time of echo (TE) = 2.96 ms, flip angle (FA) = 9◦, voxel size = 1 × 1 × 1 mm^3^. Then, a resting‐state functional magnetic resonance image scan was carried out using a single‐shot, gradient‐recalled echo planar imaging sequence with a total of 240 volumes of 54 slices covering the whole brain with the following parameters: TR = 3000 ms, TE = 24 ms, slice thickness = 3mm, flip angle = 90º, FOV = 216 × 216 mm^2^, matrix size = 72 × 72, voxel size = 3 × 3 × 3 mm^3^. During the resting‐state scanning, all participants were instructed to remain in a relaxed state without engaging in cognitive or motor activity and to keep their eyes closed. Additionally, HCs completed only one MRI session using the identical imaging parameters.

### 2.5 MRI data preprocessing

For each participant, resting‐state fMRI images were preprocessed according to the following steps. The first 10 functional scans were discarded to eliminate transients and account for T1 relaxation effects. The remaining functional images were preprocessed using standard protocols in FSL v6.0 (FMRIB’s Software Library, www.fmrib.ox.ac.uk/fsl) and included the following steps: (1) Slice timing was performed to compensate for acquisition delays across slices; (2) motion artifacts of timing corrected images were estimated and corrected by realigning all functional images to the middle image; (3) linear‐spatial normalization to MNI space by using unified segmentation of the high‐resolution T1‐weighted anatomical image; (4) voxel re‐sampling to 2 × 2 × 2 mm^3^ resolution; (5) spatial smoothing with a 6mm full‐width‐half‐maximum (FWHM) Gaussian kernel; (6) band‐pass temporal filtering (0.01–0.1Hz): removal of very low (<0.01Hz)‐ and high‐frequency (>0.1Hz) band with a finite impulse response filter, which was reported to be of physiological importance; (7) nuisance signal regression: The time series of nuisance signals were regressed out in the functional connectivity analyses, including six head motion parameters and three averaged signals representing white matter (WM), cerebrospinal fluid (CSF), and global signal, and the first temporal derivatives of aforementioned parameters. The six head motion parameters were obtained from the motion correction preprocessing step, expressed as absolute differences from the middle time point in each of the three translational and rotational directions. To extract the nuisance covariate time series for the WM, CSF, and global signal, each individual's high‐resolution T1‐weighted anatomical image was segmented by using FSL’s FAST segmentation program. The resulting segmented WM and CSF images were then threshold to ensure 80% tissue type probability. The mean time series was calculated by averaging across all voxels within the threshold masks among each individual's time series.

### 2.6 PAG seed‐based functional connectivity analysis

A seed‐region approach was conducted to perform the functional connectivity analysis, using the right ventral PAG (x = 4, y = −26, z = −14, with 3 mm radius) as the functional connectivity seed (see Figure S1). The rationale for choosing this location as a seed in our study were as follows: (1) Previous study has found that it acts a vital role in opioid antinociception^36^; (2) previous research has found the involvement of this region in pain regulation^37^; and (3) this region has been adopted in many previous pain‐related studies.^38^, ^39^, ^40^ Additionally, we also used the left PAG (x = −4, y = −26, z = −14, with 3 mm radius) as a control seed region (see Figure S1). However, there was no significant difference observed in left PAG (a control seed region)‐based functional connectivity between groups. Thus, in this study, all results are about right ventral PAG‐based functional connectivity analysis.

PAG‐based resting‐state functional connectivity was performed by using custom MATLAB scripts. For each participant, time series was the averaged time course of all voxels within PAG seed region. Then, Pearson's correlation coefficients between the PAG seed's time series and time series of every voxel across the whole brain were calculated. These resulting correlation coefficients were later transformed to Z‐scores using Fisher's transformation. Fisher's Z‐scores of correlation coefficients were expressed as functional connectivity strength.

Group level two‐sample unpaired *t*‐test was firstly performed to compare functional connectivity based on PAG seed between patients with CNSP at pre‐acupuncture and HCs. Then, two‐sample paired *t*‐test was conducted between patients with CNSP at pre‐acupuncture and at post‐acupuncture. Functional connectivity statistical maps were defined using non‐parametric permutation testing, thresholded using the threshold‐free cluster enhancement (TFCE) method, and corrected for multiple comparisons with a family‐wise error (FWE) rate of *P <* 0.05. Additionally, the effects of age and group differences of neuropsychological measures were controlled as covariates.

Finally, two‐sample *t*‐tests were analyzed to examine modulation effect of acupuncture treatment on PAG‐based functional connectivity strength. We firstly use two‐sample paired *t*‐test to investigate functional connectivity strength difference between pre‐acupuncture and post‐acupuncture in patients with CNSP. Then, two‐sample unpaired *t*‐tests were conducted to investigate functional connectivity strength difference between patients with CNSP at pre‐acupuncture and HCs, as well as between patients with CNSP at post‐acupuncture and HCs. A threshold of *P <* 0.05 was considered statistically significant after Bonferroni corrections.

### 2.7 Statistical analysis

Statistical analyses of neuropsychological assessments were performed in SPSS v25.0. The Lilliefors tests were conducted for all variables to ensure they were normally distributed. For the data that failed normality testing, nonparametric test was used. The demographic characteristics were compared between patients and HCs using two‐sample independent *t*‐test for age and education level. Two‐sample paired *t*‐tests were performed to investigate whether patients with CNSP would exhibit better improvement at post‐acupuncture compared to the performance at pre‐acupuncture related to the assessments about neck functionality. For cognitive and clinical symptoms measures, we firstly use two‐sample paired *t*‐test to investigate difference between pre‐acupuncture and post‐acupuncture in patients with CNSP. Then, two‐sample unpaired *t*‐tests were conducted to investigate difference between patients with CNSP at pre‐acupuncture and HCs, as well as between patients with CNSP at post‐acupuncture and HCs. A threshold of *P <* 0.05 was considered statistically significant after Bonferroni corrections.

Additionally, spearman correlation analyses between increased PAG‐based functional connectivity strength and improved neuropsychological assessments in patients after acupuncture treatment with the effect of age controlled as covariate were conducted to furtherly explore whether the enhanced PAG‐based functional connectivity after acupuncture treatment was related to the improvement of clinical symptoms following patients with CNSP. A significance level was set at *P <* 0.05 after Bonferroni corrected for multiple comparisons.

Before the correlation analyses, the percentage change of the functional connectivity strength in patients was calculated as described below:

$${\text{PAG ‐ based ‐ FC}}_{\text{PercentChange}}=\left[\frac{\left({\text{PAG ‐ based ‐ FC}}_{\text{post}}‐{\text{PAG ‐ based ‐ FC}}_{\text{pre}}\right)}{{\text{PAG ‐ based ‐ FC}}_{\text{pre}}}\right]\times 100\%$$

where PAG‐based‐FC_PercentChange_ is the percent change of the PAG‐based functional connectivity strength between pre‐acupuncture and post‐acupuncture in patients. The percent change of the improved neuropsychological assessments in patients was calculated as the same as the functional connectivity strength.

## 3 RESULTS

### 3.1 Demographic and neuropsychological measures

There were no significant differences in age and education level between patients with CNSP and HCs (all *P >* 0.05). Compared to the scores at pre‐acupuncture, patients with CNSP exhibited significant improvement in NRS (*t*_(1,29)_ = 16.687, *P <* 0.001), PCS (*t*_(1,29)_ = 2.910, *P <* 0.01), NPQ (*t*_(1,29)_ = 10.575, *P <* 0.001), and NDI (*t*_(1,29)_ = 9.528, *P <* 0.001) scores at post‐acupuncture (Table 1).

**TABLE 1:** Demographic characteristics and neuropsychological measures in patients with CNSP and HCs

Demographic characteristics	Patients with CNSP (*n* = 30)	HCs (*n* = 30)
Handedness (L/R)	0/30	0/30
Sex (M/F)	0/30	0/30
Age (years)	42.33 (13.16)	46.23 (14.68)
Education level (years)	15.20 (4.14)	14.07 (5.54)

Neuropsychological measures	Pre‐acupuncture	Post‐acupuncture	
NRS score	6.07 (1.36)	1.73 (1.28)	NA
PCS score	16.47 (13.17)	9.57 (8.05)	NA
NPQ score	43.97 (13.14)	16.44 (7.88)	NA
NDI score	37.47 (14.77)	14.97 (8.39)	NA
MoCA score	25.93 (3.18)	26.77 (3.07)	27.47 (3.00)
MMSE score	28.00 (1.78)	28.37 (1.73)	28.70 (1.78)
HAM‐A score	11.77 (8.02)	7.80 (7.29)	5.83 (6.52)
HAM‐D score	12.10 (9.00)	9.20 (7.61)	3.70 (3.91)

Note

Continuous variables are reported as mean (standard error).

Abbreviations: AM‐A, Hamilton Anxiety Rating Scale; CNSP, chronic neck and shoulder pain; F, female; HAM‐D, Hamilton Depression Rating Scale; HCs, healthy controls; L, left; M, male; MMSE, Mini‐Mental State Examination; MoCA, Montreal Cognitive Assessment; NA, not applicable; NDI, Neck Disability Index; NPQ, Northwick Park Neck Pain Questionnaire; NRS, Numerical Rating Scale; PCS, Pain Catastrophizing Scale; R, right.

Additionally, compared to pre‐acupuncture, patients with CNSP after post‐acupuncture showed significant improvement in HAM‐A score (*t*_(1,29)_ = −2.5417, *P <* 0.05, Bonferroni corrected), which showed no difference with HCs (*t*_(1,58)_ = 1.101, *P >* 0.05, Bonferroni corrected). Though patients with CNSP after post‐acupuncture exhibited significant improvement in HAM‐D score (*t*_(1,29)_ = −2.116, *P <* 0.05, Bonferroni corrected), but still showed significant higher HAM‐D score compared to HCs (*t*_(1,58)_ = 3.522, *P <* 0.05, Bonferroni corrected). Furthermore, there was no significant group differences in MMSE and MoCA scores between patients and HCs (all *P >* 0.05, Bonferroni corrected). All demographic and neuropsychological parameters are summarized in Table 1.

### 3.2 PAG‐based functional connectivity before acupuncture treatment

**FIGURE 2:**
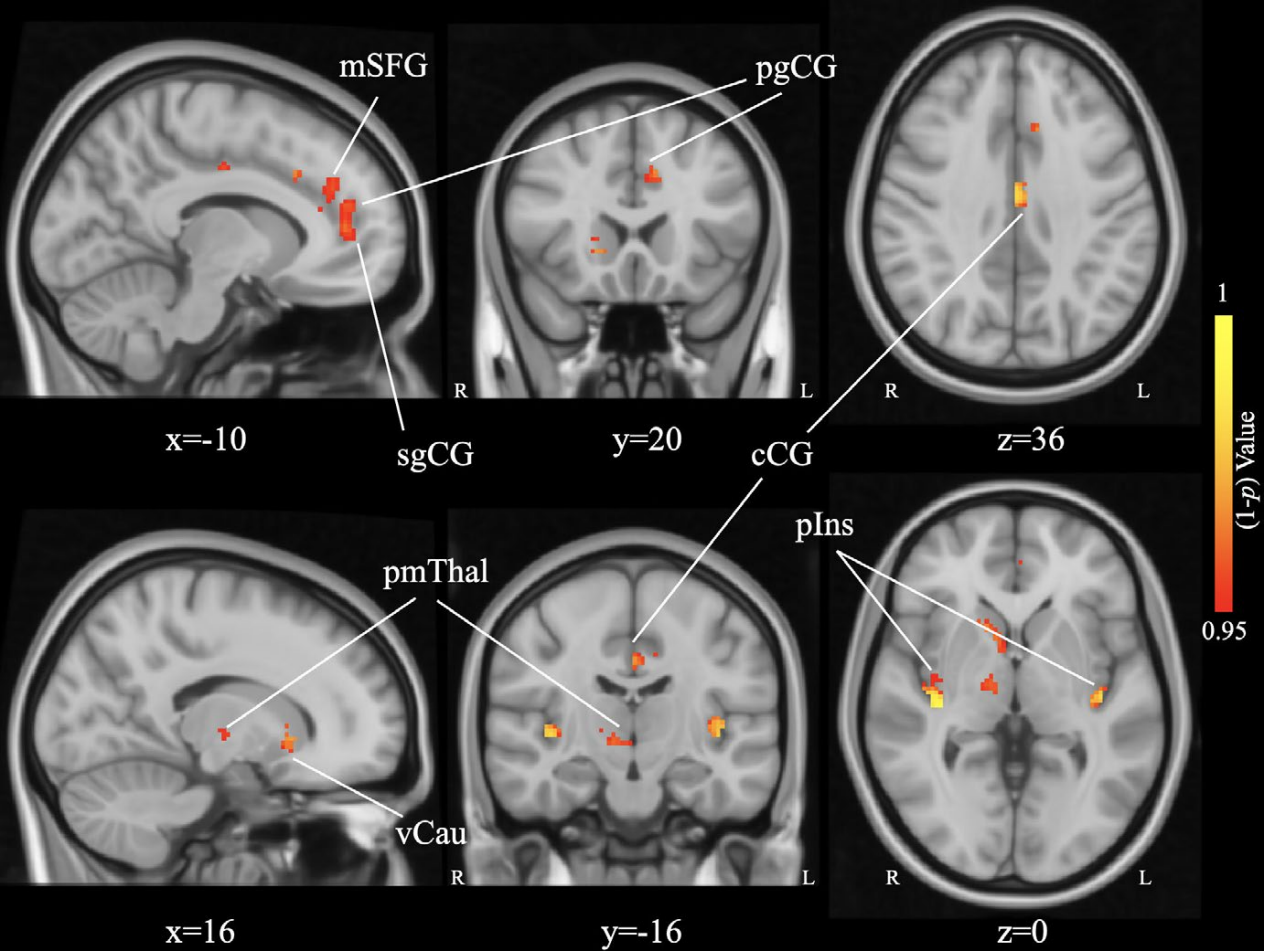
Brain regions demonstrating decreased functional connectivity with PAG seed in patients with CNSP at pre‐acupuncture compared to HCs, including the left medial Superior Frontal Gyrus (mSFG), bilateral posterior Insula (pIns), left pregenual Cingulate Gyrus (pgCG), left caudal Cingulate Gyrus (cCG), left subgenual Cingulate Gyrus (sgCG), right ventral caudate (vCau), and right pre‐motor thalamus (pmThal). The statistical threshold was *p* < 0.05, FWE corrected for all clusters. L, left; R, right

Compared with HCs, patients with CNSP demonstrated decreased PAG‐based resting‐state functional connectivity with widespread brain regions, including the left medial superior frontal gyrus, bilateral posterior insula, left pregenual cingulate gyrus, left caudal cingulate gyrus, left subgenual cingulate gyrus, right ventral caudate, and right pre‐motor thalamus (Figure 2). No clusters with increased PAG‐based functional connectivity were detected in patients with CNSP during this comparison. The coordination, peak values, and voxel size of significant clusters are summarized in Table 2.

**TABLE 2:** Brain regions demonstrating decreased functional connectivity with PAG seed in patients with CNSP at pre‐acupuncture compared to HCs

Brain region	Hemisphere	Size of cluster	Peak MNI Coordinates			P*
		(voxels)	x	y	z	
mSFG	L	178	−10	20	36	0.024
pIns	L	92	−40	−18	0	0.008
	R	66	36	−22	0	0.001
pgCG	L	84	−4	48	12	0.022
cCG	L	72	−1	−8	34	0.006
sgCG	L	85	−4	48	10	0.021
vCau	R	90	16	18	−2	0.019
pmThal	R	54	10	−16	−4	0.031

Abbreviations: cCG, caudal Cingulate Gyrus; CNSP, chronic neck and shoulder pain; HCs, healthy controls; L, left; MNI, Montreal Neurological Institute; mSFG, medial Superior Frontal Gyrus; PAG, periaqueductal gray; pgCG, pregenual Cingulate Gyrus; pIns, posterior Insula; pmThal, pre‐motor thalamus; R, right; sgCG, subgenual Cingulate Gyrus; vCau, ventral caudate.

*p < 0.05, FWE corrected.

### 3.3 PAG‐based functional connectivity after acupuncture treatment

**FIGURE 3:**
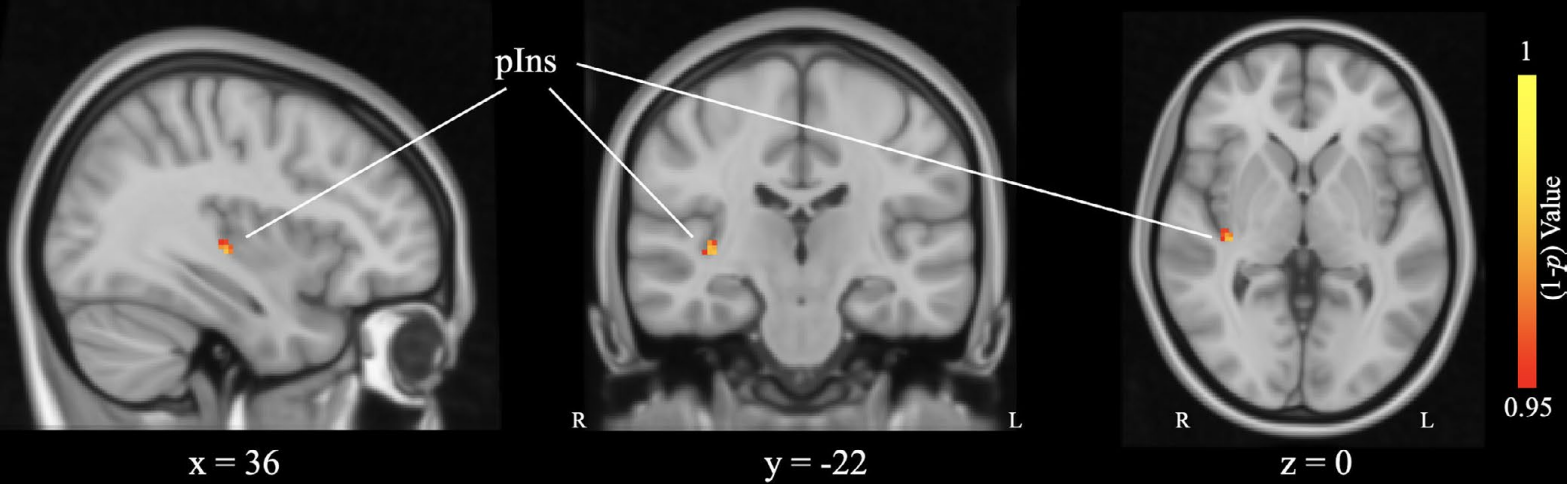
After acupuncture treatment, patients with CNSP exhibited increased PAG‐based functional connectivity with right posterior insula (pIns) compared to that before treatment. The statistical threshold was *p* < 0.05, FWE corrected for all clusters. L, left; R, right

After acupuncture treatment, patients with CNSP exhibited increased PAG‐based functional connectivity with right posterior insula (pIns) compared to that before treatment (Table 3, Figure 3).

**TABLE 3:** Brain regions demonstrating increased functional connectivity with PAG seed at post‐acupuncture compared to pre‐acupuncture in patients with CNSP

Brain region	Hemisphere	Size of cluster	Peak MNI Coordinates			P*
		(voxels)	*x*	*y*	*z*	
pIns	R	21	36	−22	0	0.002

Abbreviations: CNSP, chronic neck and shoulder pain; MNI, Montreal Neurological Institute; PAG, periaqueductal gray; pIns, posterior Insula; R, right.

*p < 0.05, FWE corrected.

**FIGURE 4:**
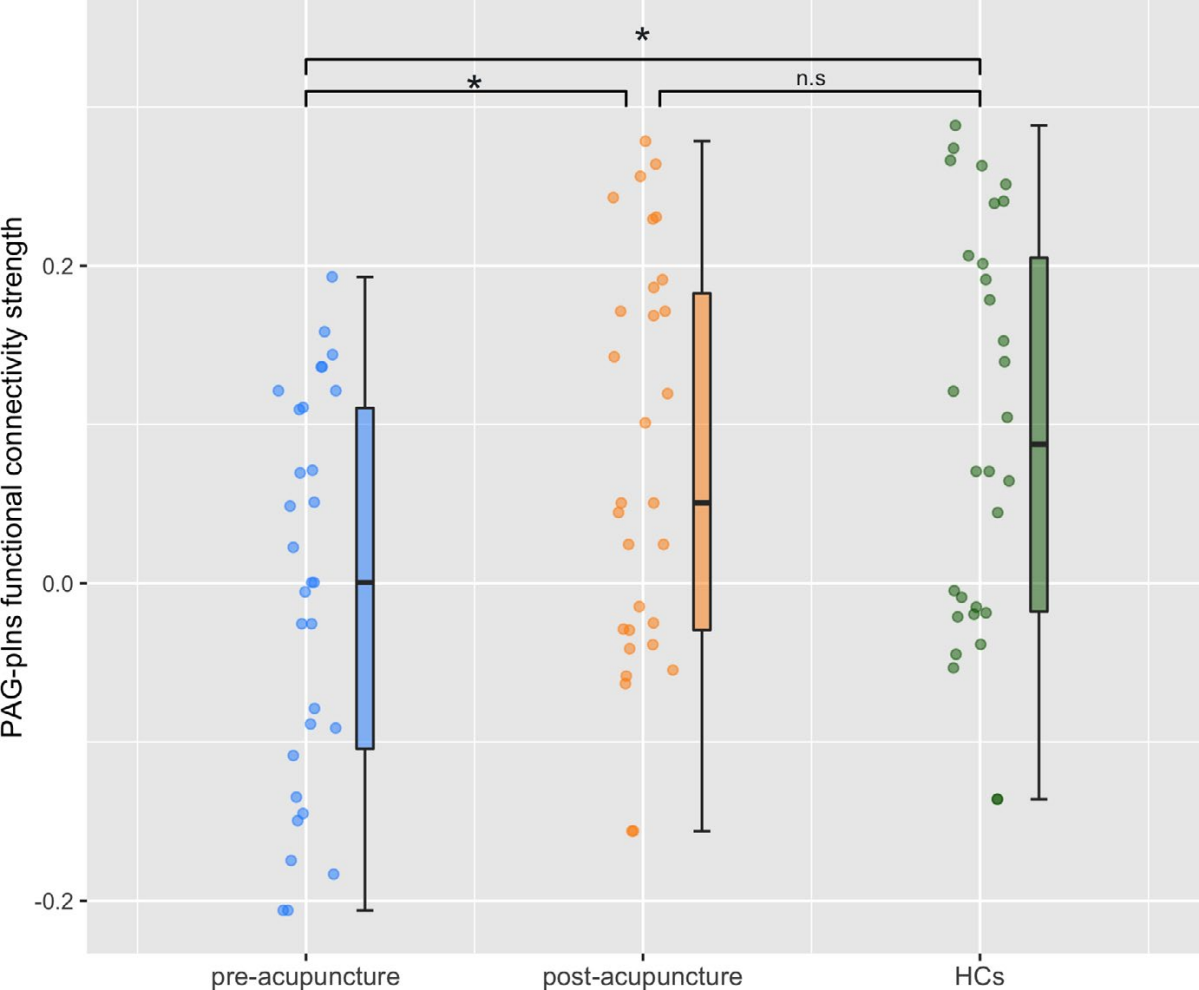
Group differences in PAG‐pIns functional connectivity strength between patients with CNSP before treatment and after treatment, and HCs. Before acupuncture treatment, patients with CNSP showed decreased PAG‐pIns functional connectivity compared to HCs, furthermore there was no significant difference in PAG‐pIns functional connectivity between HCs and patients with CNSP after acupuncture treatment. * means *p* < 0.05, Bonferroni corrected; n.s means *p* > 0.05

Furthermore, two‐sample *t*‐tests were conducted to investigate whether the increased PAG‐pIns functional connectivity strength can reflect the modulation effect of acupuncture treatment in patients. Compared to pre‐acupuncture, patients with CNSP after post‐acupuncture showed significant increased PAG‐pIns functional connectivity strength (*t*_(1,29)_ = 12.472, *P <* 0.05, Bonferroni corrected). Though before acupuncture treatment, patients with CNSP showed decreased PAG‐pIns functional connectivity strength compared to HCs (*t*_(1,58)_ = −3.079, *P <* 0.05, Bonferroni corrected), there was no significant difference in PAG‐pIns functional connectivity between HCs and patients with CNSP after acupuncture treatment (*t*_(1,58)_ = −0.591, *P >* 0.05, Bonferroni corrected, Figure 4).

### 3.4 Correlations

**FIGURE 5:**
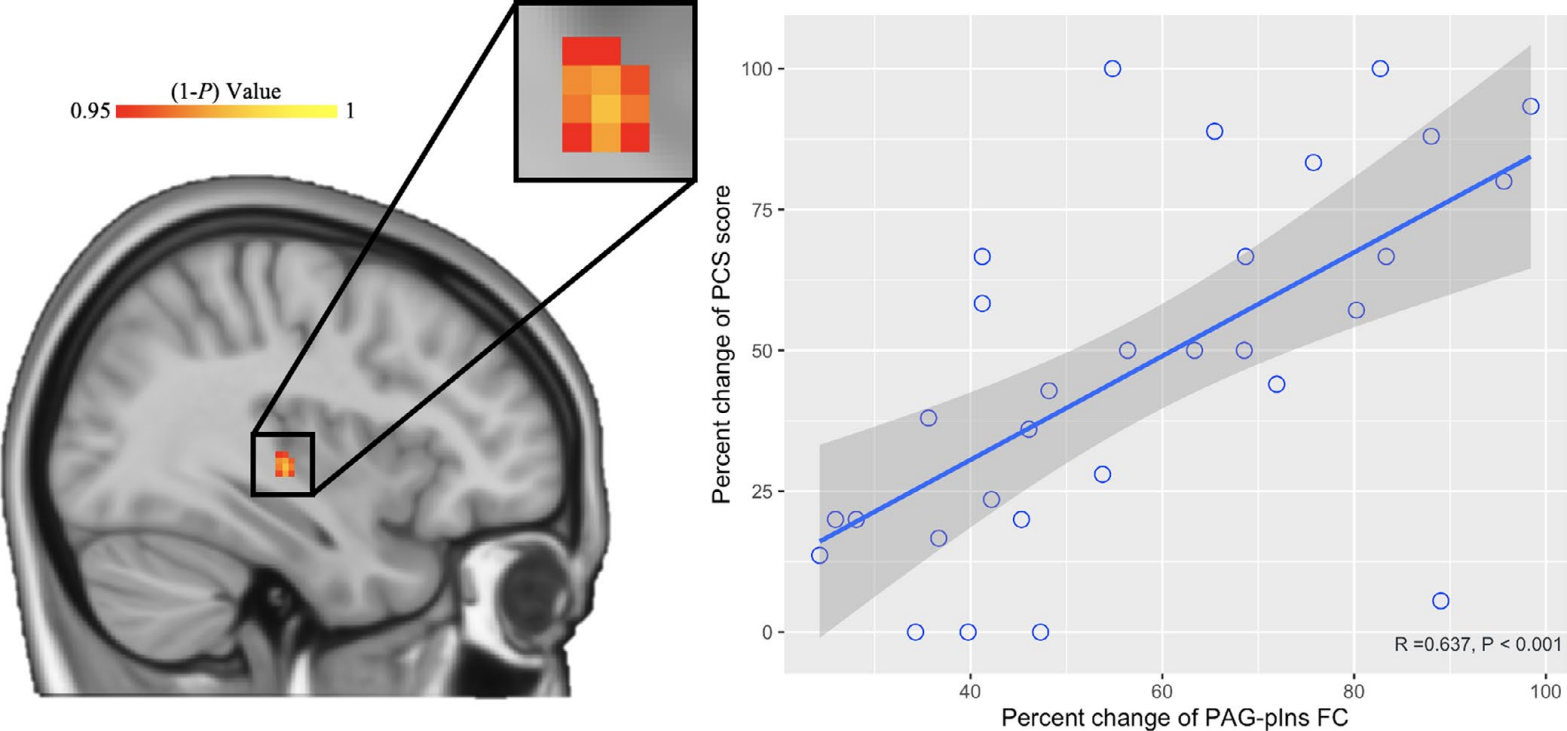
The relationship between percent change of PAG‐pIns functional connectivity (FC) strength and percent change of trait pain catastrophizing (measured by PCS) after acupuncture treatment in patients. Left panel: sagittal representation of the increased PAG‐based FC with pIns in patients with CNSP after acupuncture treatment (*p *< 0.05, FWE corrected). Right panel: the increased PAG‐pIns FC strength was significantly correlated with the decreased level of trait pain catastrophizing (measured by PCS) after acupuncture treatment (*R =* 0.637, *P <* 0.001, Bonferroni corrected)

In patients with CNSP, the increased PAG‐pIns functional connectivity strength was significantly correlated with the decreased level of trait pain catastrophizing (measured by PCS) after acupuncture treatment (*R =* 0.637, *P <* 0.001, Bonferroni corrected, Figure 5). No additional significant correlations were detected between the percent change of PAG‐pIns functional connectivity strength and any other clinical variables after acupuncture treatment.

## 4 DISCUSSION

In this study, we explored whether acupuncture treatment could be involved in normalizing PAG functional circuitry in female patients with CNSP. Before acupuncture treatment, we found that compared to HCs, patients showed altered PAG functional connectivity with widely distributed brain regions, including the left medial superior frontal gyrus, bilateral posterior insula, and cingulate gyrus. After treatment, patients with CNSP exhibited specially improved PAG‐pIns functional connectivity compared to that before treatment, and no significant difference was observed in the increased PAG‐pIns FC strength between HCs and patients with CNSP after treatment. Furthermore, pain catastrophizing reduction was significantly correlated with the increased PAG‐pIns functional connectivity strength in patients with CNSP after acupuncture treatment. Our findings suggest that acupuncture treatment may be involved in normalizing PAG functional circuitry in female patients with CNSP.

Previous research has found that PAG is a key region involved in pain modulation and thought to play an important role in the pathogenesis of chronic pain.^41^, ^42^, ^43^ Further, it has been shown that PAG seeded resting‐state functional connectivity is disrupted in chronic pain.^43^ This finding was in consist with our result, that before acupuncture treatment, patients with CNSP showed abnormal PAG‐based functional connectivity with widely distributed brain regions, including the left medial superior frontal gyrus, bilateral posterior insula, and cingulate gyrus. In rodents research investigating contextual fear discrimination, the activation of a neuronal subpopulation in the dorsal medial prefrontal cortex that projects to PAG have been found.^44^ This may explain that in our study PAG‐frontal functional connectivity altered. The superior frontal gyrus is located in the upper frontal lobe and is mainly involved in mental functions such as emotion regulation and emotional changes.^45^ It can be inferred that patients with CNSP showed severe anxiety and depression symptoms, which may result from this circuitry disrupted. Additionally, the insula is a hub region in salience network, which was mainly involved in the perception process of pain salience stimulus.^46^ The abnormal PAG‐insula functional connectivity in our findings may prove that patients with CNSP suffer chronic pain stimulus and related perception ability impaired.

After acupuncture treatment, patients with CNSP exhibited significant improvement in neck functional ability and reduced neck pain and pain catastrophizing. This result was consistent with previous findings^18^ that acupuncture treatment reduced the intensity and frequency of muscle pain, and the degree of headaches in patients. Our finding furtherly proved that the modulation effect of acupuncture treatment on CNSP was evident in reducing the clinical symptoms of patients. Furthermore, we also found improved PAG‐pIns functional connectivity in patients with CNSP after treatment. In our study, acupuncture was performed until deqi sensation was obtained. Previous study has found that acupuncture was related with stronger deqi sensations,^47^ and it is possible that the acupuncture treatment in our study activated the descending pain modulation system, initiating the release of endogenous opioids, and inhibiting nociceptive signaling from the periphery,^48^ which finally resulted the PAG‐pIns functional connectivity improvement. Furtherly, PAG has been proved as a hub region involved in pain modulation system and played an important role in the pathogenesis of chronic pain.^41^, ^42^, ^43^ Additionally, the pIns is both anatomically and functionally well suited to serve a primary and fundamental role in pain processing.^28^, ^29^ Our finding may suggest acupuncture treatment had modulation effect on pain‐processing functional networks, especially the PAG‐pIns functional connectivity.

Furtherly, we found that the increased PAG‐pIns functional connectivity strength was significantly correlated with the decreased level of trait pain catastrophizing in patients after acupuncture treatment. Previous research has found that perceived painfulness could be predicted by the functional connectivity between the PAG and insula,^33^ suggesting that PAG and insula interact together to determine pain perception. Another study has reported that activation in the contralateral pIns was positively correlated with temperature level; however, subjective intensity related more to activation of the right anterior insula.^49^ The pIns has been suggested to provide a primary “interoceptive cortex,” which specialized for perception of internal bodily states incorporating pain and autonomic arousal.^49^, ^50^ We found that the reduced level of trait pain catastrophizing was significantly associated with increased functional connectivity strength between the PAG and pIns in patients with CNSP after treatment. Previous research about mind‐wandering as well as acupuncture has found distraction from pain was associated with enhanced PAG and increased insula activations,^51^, ^52^ which suggested that correlated activity between the two regions results in reduced pain sensation. What's more, acupuncture was also found to induce a higher level of correlations among the amygdala‐related networks including the PAG and insula,^53^ also suggesting that PAG‐pIns functional connectivity may be involved in pain modulation, likely by shifting attention away from pain and changing pain processing from negative events, finally resulting in reduced level of trait pain catastrophizing.^54^ In line with this, the acupuncture treatment in our study may be involved in normalizing PAG functional circuitry, which associated with reduced level of trait pain catastrophizing.

Several limitations of this study bear acknowledgment here. First, only female patients were included. Future studies may consider including both male and female patients with CNSP to explore whether the findings differ as a function of sex. Second, there were only two time points (pre‐acupuncture and post‐acupuncture) in our study design. Future studies should include more MRI scans after acupuncture treatment to better understand long‐term effect of acupuncture treatment in CNSP. Third, only functional connectivity was performed in this study, and thus, multi‐modal neuroimaging markers are needed to investigate in future studies.

## 5 CONCLUSIONS

In summary, this study revealed that after acupuncture treatment, patients with CNSP exhibited specially improved PAG‐pIns functional connectivity, and no significant difference was observed in the increased PAG‐pIns FC strength between HCs and patients with CNSP after treatment. More importantly, the increased PAG‐pIns functional connectivity strength was significantly correlated with pain catastrophizing reduction after treatment. Taken together, these findings suggested that acupuncture treatment may be involved in normalizing PAG functional circuitry in female patients with CNSP and provide new therapeutic approaches to treating CNSP conditions.

## CONFLICT OF INTEREST

The authors declare that they have no conflict of interest.

## AUTHOR CONTRIBUTIONS

All named authors meet the International Committee of Medical Journal Editors (ICMJE) criteria for authorship for this article, take responsibility for the integrity of the work as a whole, and have given their approval for this version to be published.

H.X. and C.G. designed the research; Y.C., C.X., and Y.T. performed the analyses; Y.C. and Y.T. processed the MRI data; Y.Z., T.Z., M.W., L.F., and Y.Z. collected the behavioral and MRI data; H.X., Y.C., and Y.T. wrote the paper; H.X., Y.C., Y.T., Y.Z., T.Z., C.K., M.W., L.F., Y.Z., and C.G. critically revised the paper.

## Supporting information

Fig S1

## Data Availability

The datasets generated during and/or analyzed during the current study are available from the corresponding author on reasonable request.
