# Supplementary material for: Modulation effect of acupuncture treatment on chronic neck and shoulder pain in female patients: Evidence from periaqueductal gray‐based functional connectivity
Source: CNS Neurosci Ther. 2022 Jan 19;28(5):714–23. doi: 10.1111/cns.13803 (PMC8981480; doi:10.1111/cns.13803)
Supplement: Supplementary file 1 — Fig S1 [file CNS-28-714-s001.docx]

**Supplementary Materials**


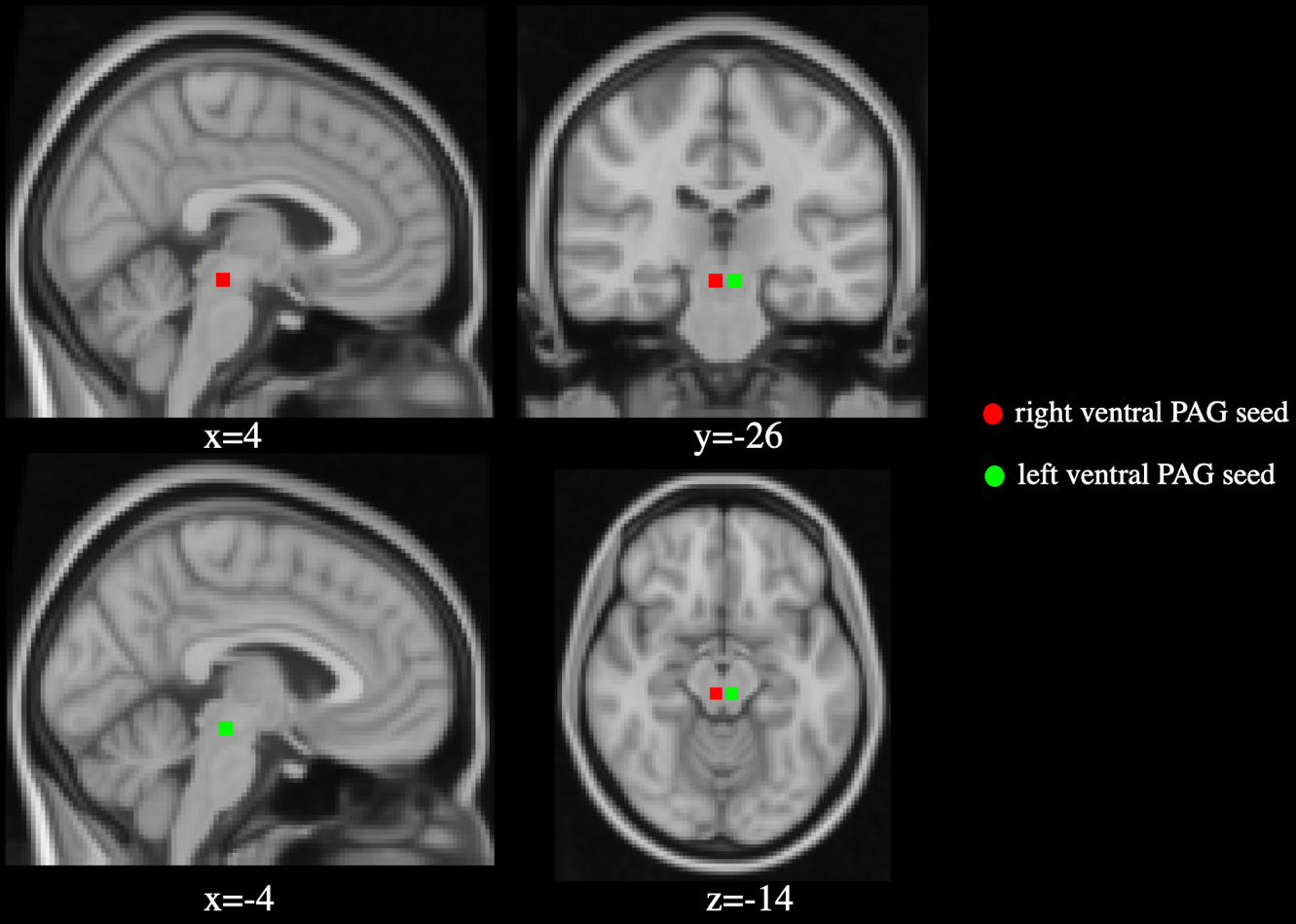


Figure S1. Representation of the PAG seeds in this study. The right ventral PAG (x = 4, y = −26, z = −14, with 3 mm radius) as the FC seed was shown in red color. The left ventral PAG (x =- 4, y = −26, z = −14, with 3 mm radius) was shown in green color as a control seed region. However, there was no group difference observed in left PAG (a control seed region) based FC between patients with CNSP before treatment and HCs. Hence in this study, all results are from right ventral PAG based FC analysis.
